# Oxidation Resistance and Wetting Behavior of MgO-C Refractories: Effect of Carbon Content

**DOI:** 10.3390/ma11060883

**Published:** 2018-05-24

**Authors:** Zhaoyang Liu, Jingkun Yu, Xin Yang, Endong Jin, Lei Yuan

**Affiliations:** School of Metallurgy, Northeastern University, Shenyang 110819, China; zhaoyangliu@stumail.neu.edu.cn (Z.L.); 18241279158@163.com (X.Y.); 1510238@stu.neu.edu.cn (E.J.); yuanl@smm.neu.edu.cn (L.Y.)

**Keywords:** oxidation resistance, wetting behavior, MgO-C refractories, carbon content

## Abstract

Various carbon contents in the MgO-C refractory were studied with respect to the oxidation resistance and the wetting behavior with slag. The bulk density, apparent porosity, cold crushing strength, oxidation rate, and mass loss rate of the fired MgO-C refractories with various carbon contents were measured and compared. The wetting and penetration behavior of the cured MgO-C refractory with the molten slag were observed in-situ. The contact angle and the shape parameters of molten slag, including the apparent radius, height, and volume were compared. The results showed that the regenerated MgO effectively restrained the carbon oxidation in the MgO-C refractory, which was more evident at the low carbon content refractory. The contact angle between the MgO-C refractory and the molten slag increased as the carbon content increased. The increased contact angle decreased the penetration of the molten slag.

## 1. Introduction

Carbon bonded magnesia (MgO-C) refractory is commonly used in the iron- and steelmaking process, especially in the lining of electric furnaces, converters, and ladles [1]. The MgO-C refractory is subjected to extreme circumstances, including ultra-high temperature, severe convection, and oxidizing atmospheres, where it becomes damaged and its service life is shortened [2,3]. Many studies have investigated the mechanism of the degradation of MgO-C refractory in slag or slag-steel systems via stationary or rotary immersion methods [4,5,6]. Jansson et al. [7] suggested that dissolution of the MgO into the slag was the first step in the corrosion of the MgO-C refractory, followed by penetration of the grain boundaries and dispersion of the grains in the slag. At the same time, the experiments results [8] showed that the mass loss of MgO-C refractory was directly dependent on the oxygen potential in the ambient atmosphere. The mechanism of MgO-C refractory degradation primarily consists of the carbon oxidation and the MgO dissolution.

Carbon plays an important role in improving the thermal shock resistance and the slag corrosion resistance of the MgO-C refractory, due to its low thermal expansion coefficient and its poor wettability with slag [9]. However, carbon is susceptible to oxidation [10], particularly in high temperatures, which is classified into direct (Equation (1)) and indirect oxidation (Equation (2)). Numerous studies have shown that many factors, such as porosity [11], reactivity of the graphite [12], gas composition, and flow characteristics [13], could dramatically affect the direct oxidation rate of the refractory. Also, the oxidation kinetics of the MgO-C refractory was studied with respect to different firing temperatures and holding times. Most studies used a lower temperature (≤1400 °C) for investigation, in order to avoid the indirect reaction, or only studied the single carbon content of the MgO-C refractory [14,15,16]. In general, the service temperature of the MgO-C refractory is typically higher than 1500 °C, thus it is necessary to study the oxidation behavior of the MgO-C refractory with various carbon contents at higher temperatures.

2C(s) + O_2_(g) = 2CO(g) (1)

C(s) + MgO(s) = CO(g) + Mg(g)(2)

The wettability characteristics between molten slag and refractories are an important indicator for the slag corrosion resistance of the refractory, which is characterized by the contact angle between the solid–liquid phases [17,18]. In general, the refractories have superior slag corrosion resistance when they have poor wettability with molten slag [19]. Heo et al. [20] suggested that the carbon in the MgO-C refractory effectively hindered the penetration of slags by repelling the slag and slowing the diffusion of Mg^2+^. Shen et al. [21] investigated the wettability between the MgO-C substrate and the ladle furnace (LF) refining slag, where they found that the MgO-C substrate remained un-wetted by the molten slag when the temperature was below 1460 °C. In fact, the wettability between MgO-C refractories and molten slag depends on many factors, such as the carbon content, temperature, slag composition, and porosity of the MgO-C refractories [22,23]. However, there have been few efforts to investigate the effects of these factors on the slag corrosion resistance of the MgO-C refractory through altering its wettability with slag [24,25]. 

The oxidation resistance and the wettability characteristics were two vital factors regarding the service life of the MgO-C refractory. However, few studies have combined the two aspects to investigate the effects that the carbon content has on the performance of the MgO-C refractory. In this paper, the oxidation experiments of the MgO-C refractories with various carbon contents were studied under different firing temperatures. The bulk density, apparent porosity, cold crushing strength (CCS), and oxidation rate of the fired MgO-C refractories were measured and compared. Also, the contact angle between the molten slag and the MgO-C refractories with various carbon contents were observed in-situ under an Ar atmosphere, and the wetting and penetration process of the molten slag within the MgO-C refractories were analyzed.

## 2. Experimental

### 2.1. MgO-C Refractory Preparation

The materials used for the preparation of the MgO-C refractory were magnesia with sizes of 1–3 mm, 0–1 mm, and <0.088 mm, as well as flake graphite. The liquid resin and the aluminum metal powder were used as binders and anti-oxidant. Various compositions were formulated by varying the graphite content; see Table 1 for details. The raw materials were mixed for homogeneity, then pressed into a 50 mm cylindrical mold under a pressure of 200 MPa. The cylindrical MgO-C refractories were cured at 200 °C for 24 h. 

### 2.2. Testing and Characterization Methods

The oxidation resistance experiment was performed by firing the cured MgO-C refractories in an electric resistance furnace (manufacturer, city, state, country). The MgO-C refractories were heated in the furnace to the specified temperature (1400 °C or 1600 °C) for 3 h under an oxidizing atmosphere. The bulk density and the apparent porosity of the MgO-C refractories were measured by via the Archimedes principle and calculated with Equations (3) and (4).

(3) $$D=\frac{m_{0}D_{l}}{m_{2}-m_{1}}$$

(4) $$q=\frac{m_{2}-m_{0}}{m_{2}-m_{1}}$$

where *D* and *q* are the bulk density (g∙cm^−3^) and the apparent porosity (%) of the MgO-C refractories, respectively. *m*_0_ is the mass of refractories in the air (g), *m*_1_ is the mass of the refractories in the water (g), *m*_2_ is the mass of the refractories with free bubbles on the surface (g), and *D*_l_ is the density of the water (1.0 g∙cm^−3^).

The CCS of the MgO-C refractories was measured by using a compression testing machine (Constant Hydraulic Machinery Co. LTD., Zaozhuang, China) with a constant load rate, and the results were calculated with Equation (5). The mass, un-oxidized diameter, and height of the MgO-C refractory of both the initial state and after the oxidation were measured, and the mass loss rate and the oxidation rate of the refractories were calculated with Equations (6) and (7).

(5) $$C=\frac{F}{A}=\frac{4F}{\pi R_{0}^{2}}$$

(6) $$r_{m}=\frac{m_{0}-m}{m_{0}}\times 100\%$$

(7) $$r_{o}=\frac{V_{0}-V}{V_{0}}\times 100\%=\frac{R_{0}^{2}h_{0}-R^{2}h}{R_{0}^{2}h_{0}}\times 100\%$$

where the *C* is the cold crushing strength, *F* is the force imposed on the samples, *A* is the sample’s area, and *r*_m_ and *r*_o_ are the mass loss rate (%) and the oxidation rate (%), respectively. *m*_0_, *V*_0_, *R*_0_, and *h*_0_ are the mass (g), volume (mm^3^), diameter (mm), and height (mm), respectively, of MgO-C refractories before oxidation. *m* is the mass (g) of MgO-C refractories after oxidation; *V*, *R*, and *h* are the volume (mm^3^), diameter (mm), and height (mm) of the unoxidized area following oxidation. 

**Figure 1:**
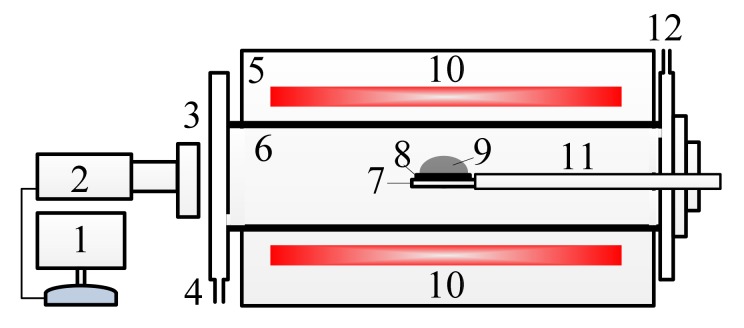
Schematic experimental setup of the wetting process. 1 Recorder, 2 High speed camera, 3 Quartz window, 4 Gas inlet, 5 Furnace, 6 Alumina tube, 7 Stage, 8 MgO-C refractory, 9 Slag, 10 Heating element, 11 Moving arm, 12 Gas outlet.

The wetting behaviors of the MgO-C refractories with molten slag were examined on the device shown in Figure 1. A 13 mm × 2 mm MgO-C disc was prepared from the above MgO-C refractory. The prepared slag was mixed and pre-melted in an inducing furnace (Shenqiu Yongda High-frequency Equipment Co. LTD., Zhoukou, China) and the chemical composition, as shown in Table 2. The temperature of the furnace was raised and maintained at 1300 °C. After the temperature was stabilized, the MgO-C refractory disc with small granule slag was send to the furnace via the moving arm. The inert atmosphere was maintained throughout the experimental process to protect the MgO-C refractory from oxidation. A high-speed camera captured and restored images of the MgO-C refractory wetting process. The shape parameters of the molten slag, such as the apparent radius and height, were measured by analyzing the images. 

## 3. Results and Discussion 

### 3.1 Oxidation Resistance

The oxidation resistance of the MgO-C refractories with various carbon contents were measured and compared by firing the refractories at 1400 °C and 1600 °C. The bulk density, apparent porosity, CCS, oxidation rate, and mass loss rate of the fired MgO-C refractories were examined.

**Figure 2:**
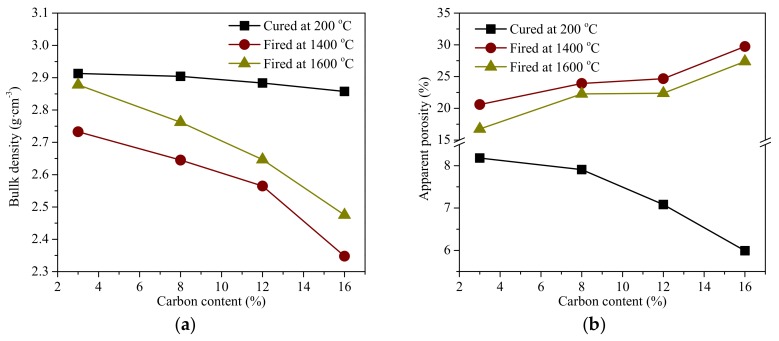
Bulk density (**a**) and apparent porosity (**b**) of the cured and fired MgO-C refractories.

Figure 2a depicts the bulk density of the cured and fired MgO-C refractories with various carbon contents. The lower density of carbon caused the bulk density of the MgO-C refractories to decrease as the carbon content increased. The bulk density of the refractories markedly decreased after firing, which was more evident with the higher carbon content. The apparent porosity of the fired MgO-C refractories increased as the carbon content increased (Figure 2b), which caused by the greater carbon oxidation present and more additional porous structures create in the higher carbon content. It is worthwhile to mention that the 1600 °C batch obtained higher bulk density and lower apparent porosity of the fired refractories.

**Figure 3:**
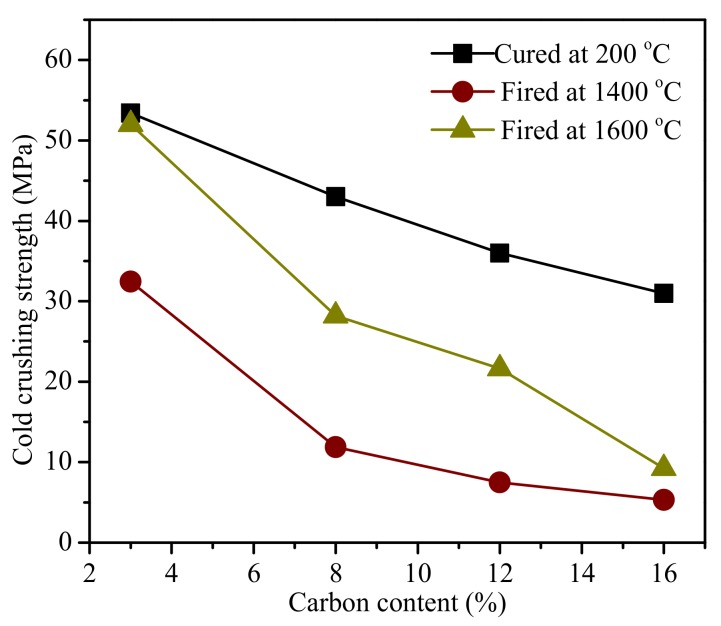
Cold crushing strength of the cured and fired MgO-C refractories.

Figure 3 shows the CCS values of the cured and fired MgO-C refractories. With the same tendency of the bulk density are shown in Figure 2a, where the CCS value decreased as the carbon content increased in the three batches, and the 1600 °C batch had a more moderate value than the other two batches. The CCS failure of the fired MgO-C refractories, especially in higher carbon content, was due to the abundant pores that were generated by the carbon oxidation, which made the composition weak and fragile. The changed trends of CCS were in agreement with those reported [12] by other researchers. 

**Figure 4:**
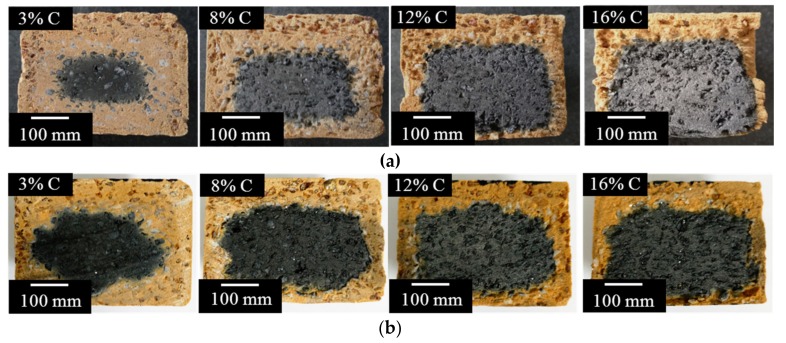
Longitudinal sections of the fired MgO-C refractories at: (**a**) 1400 °C and (**b**) 1600 °C.

**Figure 5:**
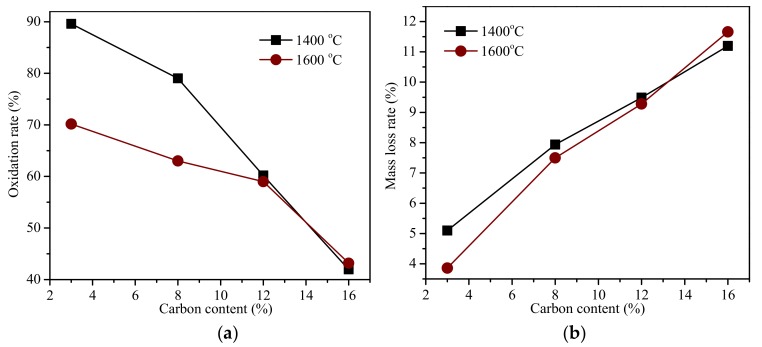
Oxidation rate (**a**) and mass loss rate (**b**) of the fired MgO-C refractories.

Figure 4 shows the longitudinal section of the fired MgO-C refractories under two different firing temperatures. The boundary between the non-oxidized core and the completely oxidized exterior shell was present, and the oxidized exterior shell decreased as the carbon content increased both of the two batches. The volumes of the original and the non-oxidized core after oxidation were calculated and the oxidation rates are shown in Figure 5a. When the carbon content of MgO-C refractories were 3% and 8%, the oxidation rate of the 1600 °C batch was obviously smaller than the 1400 °C batch, while the two batch values were became almost the same when the carbon content were raised to 12% and 16%. The mass loss rate of the fired MgO-C refractories is show in Figure 5b. There were differences within the trends of oxidation rates, which increased as the carbon content increased. 

The carbon oxidation of MgO-C refractory fired at 1400 °C was direct oxidation (Equation (1)). However, the situation became more complicated when fired at 1600 °C. The carbon oxidation of the MgO-C refractory was the comprehensive result of the direct (Equation (1)) and the indirect (Equation (2)) oxidation. The indirect oxidation reaction of the carbon generated Mg vapor, which diffused outside, through the porous structure of MgO-C refractory, where it came in contact with oxygen and reoxidized to MgO (Equation (8)). The regenerated MgO filled up the pores and impeded additional oxygen from entering, furthering the oxidation to some extent. Therefore, the oxidation was restrained in the 1600 °C batch, which was seen in the longitudinal section morphology of the fired MgO-C refractory (Figure 4). The indirect oxidation effects were also reflected in the other parameters, such as the higher density and CCS value, and lower porosity and mass loss rate of the fired refractory at 1600 °C. As the carbon content increased, more porous structures were generated after the carbon oxidation. The pores filled with the deposited MgO were limited under the high carbon content of MgO-C refractory, which caused the temperature effect to be more pronounced when the carbon content was lower. Similar results were found for MgO-C composite containing 5 wt % [26] and 20 wt % [27] carbon in previous study. In their investigation, the oxidation rates were decreased when the temperature exceeded 1400 °C. They explained that the formation of a continuous dense layer on the outer brick surface restricted the oxidation of low carbon containing MgO-C refractory. 

2Mg(g) + O_2_(g) = 2MgO(s) (8)

### 3.2 Wetting Behavior

**Figure 6:**
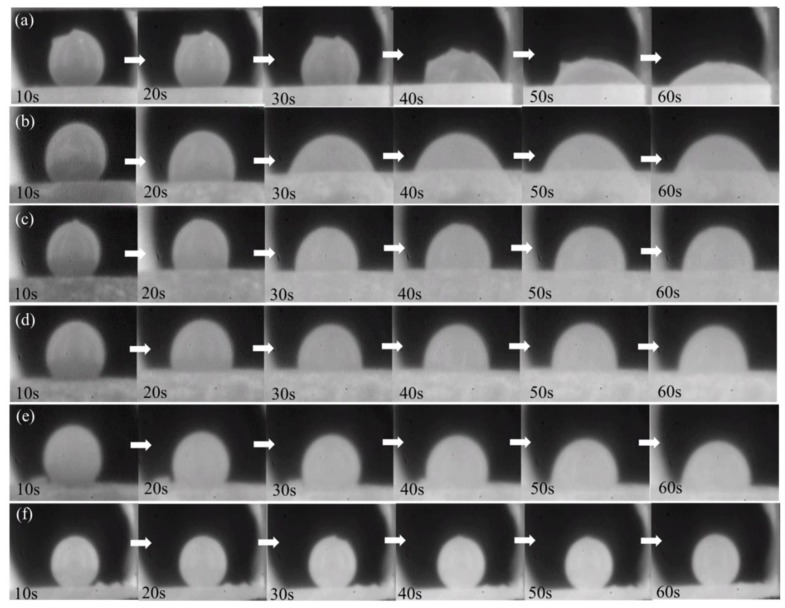
Wetting process of MgO-C refractories with the molten slag: (**a**) pure MgO, (**b**) 3% C, (**c**) 8% C, (**d**) 12% C, (**e**) 16% C, and (**f**) pure graphite.

**Figure 7:**
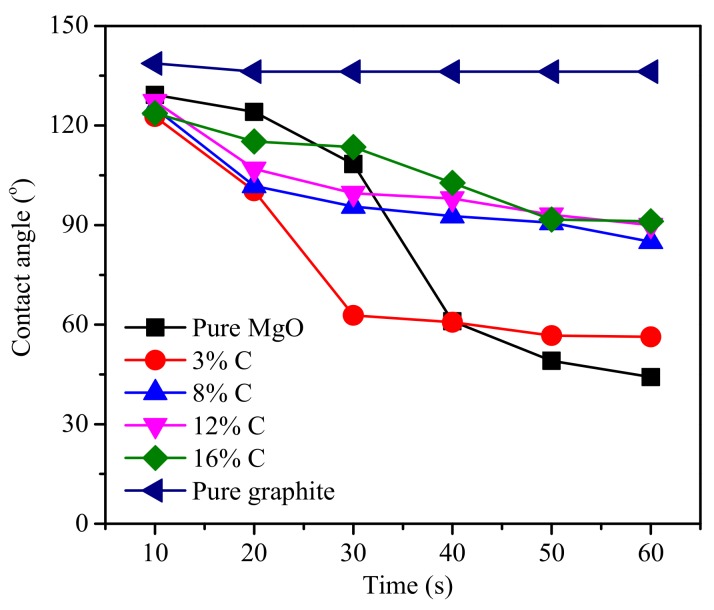
Contact angle between the samples and the molten slag.

Figure 6b–e show the wetting process of the various carbon contents of the MgO-C refractories with molten slag at 1300 °C. The wetting behavior of molten slag in the pure MgO (Figure 6a) and the graphite (Figure 6f) disc were studied for comparisons with the MgO-C refractories. The contact angle between the samples and the molten slag was measured; the results are shown in Figure 7. 

The molten slag spread to the surface of sample materials and the contact angles between them decreased as the wetting process progressed (Figure 6 and Figure 7). The contact angle between the molten slag and the pure carbon was about 140° and changed little with time, while the situation was totally different when it came to the pure MgO and MgO-C refractories. The contact angle between molten slag and pure MgO fiercely decreased from 129.2° to 61.0° within 30 s, and decreased from 122.7° to 62.8° within 20 s for the refractory with the 3% carbon content. The decrease of the contact angle was not as abrupt when the carbon content were 8%, 12%, and 16%, where there was little difference in the three different carbon content within the 60 s. Yuan et al. [22] studied the wettability between molten slag and MgO-C refractories. In their study, the contact angle reached the steady state needs longer time (237 s). It is mainly due to the high basicity (C/S = 3.3) and high carbon containing MgO-C refractories (C% ≥ 18%) they used.

**Figure 8:**
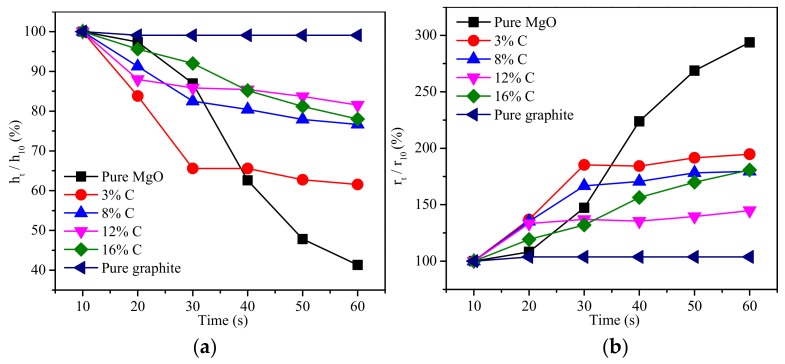
Changes of the apparent height (**a**) and the apparent radius (**b**) of the molten slag.

**Figure 9:**
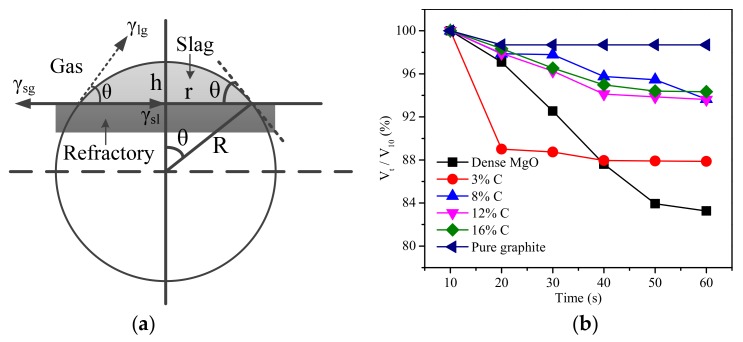
Crown model (**a**) and calculated apparent volume (**b**) of the molten slag.

The error that was caused by the slightly quality differences of the molten slag within the different samples was eliminated by using the ratio to the shape parameters (apparent radius and height) of molten slag at 10 s (Figure 8). The apparent volume of the molten slag was calculated according to the crown model (Figure 9a and Equation (9)), and the ratio of volume (ratio to the value at 10 s) is shown in Figure 9b. The change of apparent volume was strongly associated with the carbon content. For example, the apparent volume was changed slightly when the molten slag came in contact with pure carbon, while it was markedly decreased when the molten slag came in contact with the pure MgO and the refractory with 3% carbon content. When the carbon content in the composition was between 8% and 16%, the apparent volume change over time was basically the same and the values were between the pure carbon and the 3% carbon content.

(9) $$V=\frac{1}{3}\pi \left[2R^{3}(1-\cos\theta )-r^{2}(R-h)\right]$$

(10) $$R=\frac{r^{2}+h^{2}}{2h}$$

(11) $$\theta =\sin^{-1}\frac{2rh}{r^{2}+h^{2}}$$

where *V* is the apparent volume of molten slag, *r* is the apparent radius, and *h* is the apparent height; *θ* is the contact angle of between the molten slag and the MgO-C refractory; and *R* is the crown diameter. *R* and *θ* are the functions of *r* and *h*, which were calculated from Equations (10) and (11) [17].

**Figure 10:**
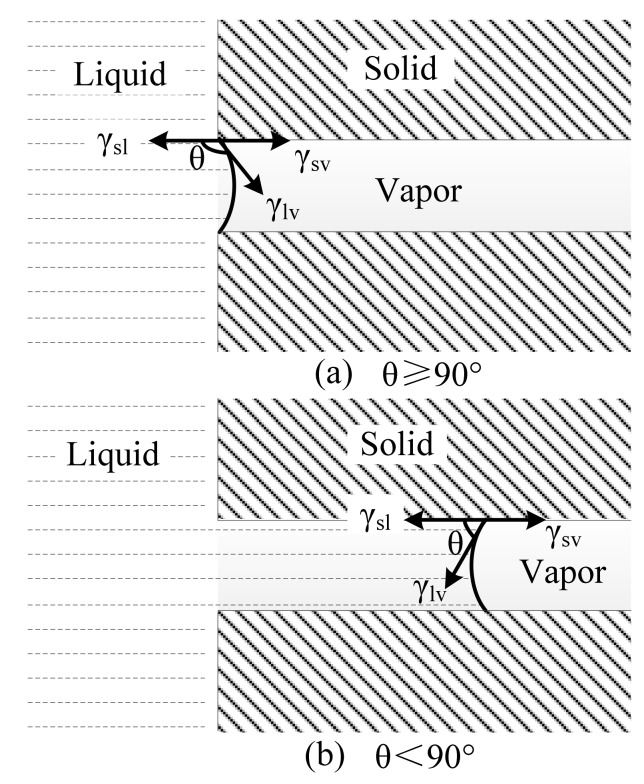
Schematic diagram of the relationship between the contact angle and the penetration (**a**) θ ≥ 90°; and (**b**) θ < 90°.

The decreased apparent volume over time suggested that the molten slag spread while it penetrated into the refractories. The extent of penetration within the molten slag into refractory was expressed by the absorption force, which was determined by the surface tension between liquid and vapor, the contact angle, and the capillary radius (Equation (12)) [24,28]. There was only a very short time at the start of the melting that was detected, it was assumed that the composition of the slag was unchanged. Therefore, the surface tension between liquid and vapor was basically unchanged. At the same time, the porosity of different refractories mostly ranged between 6% and 8% (Figure 2b). So, the absorption force had the highest correlation with the contact angle. In fact, Figure 7 and Figure 9b illustrate the close relationship between the change of contact angle and apparent volume. The smaller contact angle corresponded the reduced apparent volume, which indicated a deeper penetration of the molten slag into the refractory (Figure 10). Shen et al. [21] studied the LF refining slag and MgO-C substrate, and also claimed that the molten slag penetrated into the substrate once the contact angle was smaller than 90°.

Δ*P* = (2*γ*_lv_·cos*θ*)/*r* (12)

where Δ*P* is the absorption force of the capillary phenomena, *γ*_lv_ is the surface tension of the liquid-vapor, *θ* is the contact angle, and *r* is the radius of capillary.

## 4. Conclusions

The oxidation resistance of the MgO-C refractory was studied by comparing the bulk density, apparent porosity, cold crushing strength, and oxidation rate of the fired refractories with different carbon contents. The wetting behavior of the MgO-C refractory with molten slag was observed in-situ, and the contact angle and apparent volume were compared. The results were as follows:

1. The bulk density, apparent porosity, and cold crushing strength of the cured MgO-C refractory decreased as the carbon content increased. These properties degraded after firing, especially at higher carbon content.
2. The regenerated MgO in the MgO-C refractory effectively hindered the carbon oxidation in the lower carbon content, and increased the cold crushing strength and bulk density of the fired refractory to some extent.
3. The molten slag penetration into the MgO-C refractory, which decreased the apparent volume during the wetting process. The penetration extent was closely related to the contact angle between the MgO-C refractory and the molten slag.

## Figures and Tables

**Table 1:** Composition of the MgO-C refractories (mass%).

Material	1	2	3	4
MgO	97	92	88	84
Carbon	3	8	12	16
Al metal powder	+2	+2	+2	+2
Liquid resin	+4	+4	+4	+4

**Table 2:** Chemical composition of the experimental slag (mass %).

CaO	SiO_2_	Al_2_O_3_	V_2_O_5_	Fe_2_O_3_	MnO	TiO_2_
45.87	35.52	13.65	1.90	1.68	0.72	0.66
